# Prognostic Value of Changes in Preoperative and Postoperative Serum CA19-9 Levels in Gastric Cancer

**DOI:** 10.3389/fonc.2020.01432

**Published:** 2020-08-18

**Authors:** Xiao-Hai Song, Kai Liu, Shi-Jie Yang, Wei-Han Zhang, Xiao-Long Chen, Lin-Yong Zhao, Xin-Zu Chen, Kun Yang, Zong-Guang Zhou, Jian-Kun Hu

**Affiliations:** ^1^Department of Gastrointestinal Surgery, West China Hospital, Sichuan University, Chengdu, China; ^2^Laboratory of Gastric Cancer, State Key Laboratory of Biotherapy, Collaborative Innovation Center for Biotherapy, West China Hospital, Sichuan University, Chengdu, China; ^3^Laboratory of Digestive Surgery, State Key Laboratory of Biotherapy, Collaborative Innovation Center for Biotherapy, West China Hospital, Sichuan University, Chengdu, China

**Keywords:** gastric cancer, CA19-9, prognosis, gastrectomy, overall survival

## Abstract

**Objective:** The prognostic significance of serum CA19-9 levels in gastric cancer patients remains a matter debate. The aim of this study was to determine the prognostic value of changes in preoperative and postoperative serum CA19-9 levels in patients with gastric cancer.

**Methods:** A total of 1,046 gastric cancer patients who underwent curative gastrectomy in West China Hospital of Sichuan University from January 2011 to December 2016 were analyzed retrospectively. Patients were categorized by minimum *P*-value using X-tile, while the baseline confounders for CA19-9 changes were balanced through propensity score matching (PSM). The relationships between CA19-9 changes and other clinicopathologic features were measured. Univariate and multivariate analysis were performed to explore the risk factors associated with survival outcomes.

**Results:** We included 653 patients. Changes in CA19-9 levels significantly correlated with age, tumor size, macroscopic type, histological grade, T stage and TNM stage. Kaplan–Meier curves revealed that patients with CA19-9 changes <20% had significant better overall survival than those with changes more than 20% (*p* < 0.001); Cox regression analysis revealed the CA19-9 change (*p* = 0.010), gender (*p* = 0.031), histological grade (*p* = 0.036) and TNM stage (*p* < 0.001) were independent risk factors for survival after PSM. Stratification analysis indicated that patients with CA19-9 change more than 20% had worse prognosis that those with CA19-9 change no more than 20% in male (*p* = 0.002), poorly differentiated or undifferentiated type (*p* = 0.031) and TNM stage III (*p* = 0.006).

**Conclusion:** Changes in preoperative and postoperative serum CA19-9 levels were closely associated with clinicopathological traits and was an independent prognostic factor in gastric cancer patients. This parameter may be a reliable marker for prediction of survival.

## Introduction

Gastric cancer is one of the most common form of malignancy in China, and it is the second leading cause of cancer deaths, following lung cancer (1, 2). In China, most patients have already evolved into advanced gastric cancer by the time of diagnosis (3, 4). The standard treatments worldwide for advanced gastric cancer are surgery and systemic chemotherapy; however, the prognosis of gastric cancer patients remains poor (5, 6). The most common causes of death for gastric cancer patients after radical surgery are local recurrence and distant metastasis. It is essential to determine the prognostic factors and perform appropriate therapeutic strategies for patients with advanced gastric cancer. Among various clinicopathological factors, the depth of tumor invasion and lymph node metastasis have been identified as the most important prognostic factors in gastric cancer (7, 8).

Among the available tumor markers, carbohydrate antigen (CA) 19-9 is one of the commonly used tumor markers for patients with gastrointestinal malignancies (9, 10). Carbohydrate antigen (CA) 19-9 was identified by Koprowski et al. as an anti-sialyl-lea sugar chain antigen (11). Since then, it has been used extensively as a serum tumor marker for the diagnosis and monitoring of pancreatic cancer and gastric cancer (10, 12). In addition, some studies have shown that CA19-9 levels predicts prognoses in gastric cancer (10, 13, 14). Nevertheless, it remains unclear as to whether serum CA19-9 levels are prognostic indicators in patients with gastric cancer.

Most published studies focus only on preoperative or postoperative values of serum CA19-9 (15, 16). The prognostic value of preoperative and postoperative serum CA19-9 change has not been reported and remain unknown at present. Therefore, in this study, we measured changes in serum CA19-9 levels in patients with gastric cancer before and after curative treatment to understand its significance.

## Materials and Methods

### Ethics

Patient records were de-identified and anonymized prior to analysis. The Research Ethics Committee of West China Hospital approved this retrospective study and Surgical Gastric Cancer Patient Registry number was (No. WCH-SGCPR-2019-11).

### Patients

We retrospectively enrolled patients who were diagnosed with primary gastric cancer and underwent curative gastrectomy from January 2011 to December 2016 in the Department of Gastrointestinal Surgery, West China Hospital, Sichuan University. The following inclusion criteria were applied: (1) histologically confirmed adenocarcinoma of the stomach; (2) curative R0 according to a pathological diagnosis after surgery; (3) no preoperative treatment; (4) measurement of serum CA19-9 before and after surgery. The following exclusion criteria were applied: (1) metastatic disease; (2) preoperative treatment such as neoadjuvant chemoradiotherapy; (3) incomplete medical records; and (4) no measurement of serum CA19-9 before and after surgery. Finally, 653 patients were included.

### Data Collection

Clinicopathological traits including extent of resection, tumor location, tumor size, macroscopic type, histological grade, pathological T stage (pT stage), pathological N stage (pN stage), M stage and pathological TNM stage (pTNM stage) were recorded according to the AJCC 8th edition (17).

After undergoing gastrectomy, all patients were periodically followed up in outpatient visits and telephone interviews. The follow-up evaluation was every 3 months during the first 2 postoperative years, every 6 months in the third year, then annually thereafter. Overall survival (OS) was the primary end-point, and the survival time was calculated from the date of operation to the date of death or the last follow-up visit, in January 2019. Of the total patients, 93.3% were followed up.

### Evaluation of Changes Serum Tumor Markers

Preoperative serum levels of CA19-9 were measured within 7 days before gastrectomy, while postoperative measurement of CA19-9 levels were usually measured at the outpatient 1–4 weeks after surgery and before administration of adjuvant chemotherapy. The changes of CA19-9 level before and after surgery were labeled as change rate (α):

$$\begin{array}{l} \alpha =\text{postoperative CA}19-9-\text{preoperative}\\ \text{ CA}19-9/\text{preoperative CA}19-9 \end{array}$$

According to the X-tile plots, the optimal cutoff point for CA19-9 change rate (α) was ±.2. As a result, gastric cancer patients were divided into four groups: (1) CA19-9 decreasing more than 20% (α < -0.2); (2) CA19-9 decreasing no more than 20% (−0.2 ≤ α ≤ 0); (3) CA19-9 increasing no more than 20% (0 < α ≤ 0.2); (4) CA19-9 increasing more than 20% (α > 0.2). CA19-9 decreasing or increasing more than 20% means CA19-9 changing more than 20% (α < -0.2 or α >0.2), while CA19-9 decreasing or increasing <20% means CA19-9 changing <20% (−0.2 ≤ α ≤ 0.2).

### Statistical Analysis

X-tile program (Version 3.1.2, Yale University) was used to calculate the optimal cutoff points for the change rate (α) of each group. All statistical analyses were performed using SPSS 19.0 (SPSS®, Chicago, Illinois, USA). All continuous variables were expressed as mean ± standard deviation (SD). Unordered categorical variable and ranked data was analyzed using the chi-square test and rank sum test (Mann–Whitney *U*-test), respectively. The student's *t*-test was used to analyze continuous data if homogeneity of variance and normal distribution. If not, rank sum test was used. The Kaplan–Meier method and life-table method were used to calculate the cumulative survival rate. Log-rank test and Cox's proportional hazard regression model were applied for univariate and multivariate survival analyses, respectively. To reduce the impact of potential confounding factors and effects of selection bias such as baseline clinicopathologic factors or uneven patient distribution between the CA19-9 changing more than 20% and no more than 20%, 1:1 propensity score matching (PSM) was applied to adjust for age, tumor size, macroscopic type, histological grade, and T stage and TNM stage. A 0.2-width caliper of the standard deviation of the logit was applied to match across the two groups (18, 19). *P*-values less than 0.05 were considered significant.

## Results

### Patient Population

A total of 653 patients were included. There were 165 patients with CA19-9 decreasing more than 20% (α < −0.2), 125 patients with CA19-9 decreasing no more than 20% (−0.2 ≤ α ≤ 0), 187 patients with CA19-9 increasing no more than 20% (0 < α ≤ 0.2) and 176 patients with CA19-9 increasing more than 20% (α > 0.2); There were 341 patients with CA19-9 changing more than 20% (α < −0.2 and α > 0.2) and 312 patients with CA19-9 changing <20% (−0.2 ≤ α ≤ 0.2).

**Figure 1:**
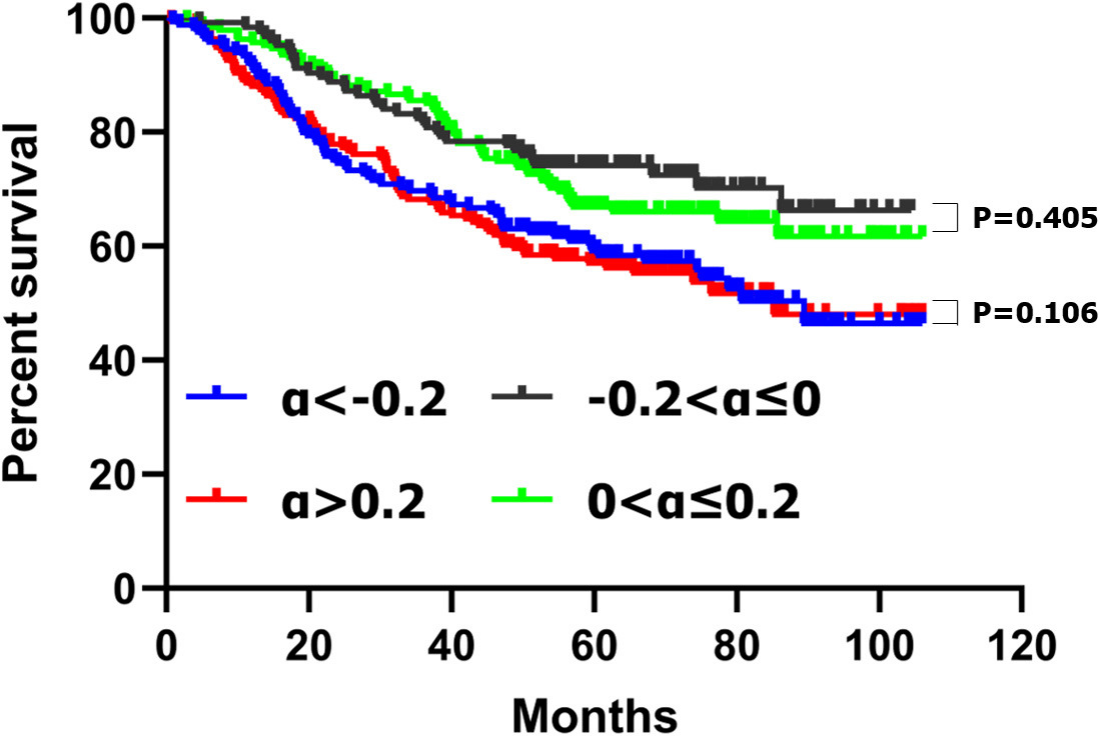
Kaplan–Meier survival analyses of four subgroups of gastric cancer patients with CA19-9 change.

There was no significant difference of the survival curves between the patients with CA19-9 decreasing no more than 20% (−0.2 ≤ α ≤ 0) and those increasing no more than 20% (0 < α ≤ 0.2) (*p* = 0.405) (Figure 1). We compared the clinicopathological features between patients with CA19-9 decreasing no more than 20% (−0.2 ≤ α ≤ 0) and those with CA 19-9 increasing no more than 20% (0 < α ≤ 0.2) and we found that there were no significant differences between the two groups in terms of tumor location, tumor size, macroscopic type, histological grade, T stage, N stage, and TNM stage (Supplementary Table 1).

A similar result was found between the patients with CA19-9 decreasing more than 20% (α < −0.2) and those with CA19-9 increasing more than 20% (α > 0.2); there was no significant difference of the survival curves (*p* = 0.106) (Figure 1) and clinicopathological features between the two groups (Supplementary Table 2).

These results suggested that patients with CA19-9 decreasing no more than 20% (−0.2 ≤ α ≤ 0) and those increasing no more than 20% (0 < α ≤ 0.2), patients with CA19-9 decreasing more than 20% (α < −0.2) and those with CA19-9 increasing more than 20% (α > 0.2) might have similar biological behaviors. Therefore, we combined patients with CA19-9 decreasing no more than 20% (−0.2 ≤ α ≤ 0) and those increasing no more than 20% (0 < α ≤ 0.2) into one group with CA19-9 change no more than 20% (−0.2 ≤ α ≤ 0.2) and combined patients with CA19-9 decreasing more than 20% (α < −0.2) and those with CA19-9 increasing more than 20% (α > 0.2) into another group with CA19-9 change more than 20% (α < −0.2 or α > 0.2); Then we compared the clinicopathological characteristics and survival of patients with CA19-9 change no more than 20% (−0.2 ≤ α ≤ 0.2) and those with CA19-9 change more than 20% (α < −0.2 or α > 0.2).

### Correlations Between Clinicopathological Findings and CA19-9 Change

Clinicopathologic features were compared between patients with the CA19-9 change no more than 20% and those with the CA19-9 change more than 20%. CA19-9 change was found to be significantly associated with age (*p* = 0.004), tumor size (*p* = 0.004), macroscopic type (*p* = 0.012), histological grade (*p* = 0.038), T stage (*p* < 0.001), and TNM stage (*p* < 0.001). Specifically, patients with CA19-9 change more than 20% were more likely to be with large tumor size, macroscopic type III–IV, deep tumor invasion, and more advanced TNM stage than patients with CA19-9 change no more than 20%. However, CA19-9 change was not significantly associated with gender (*p* = 0.353), extent of resection (*p* = 0.785), tumor location (*p* = 0.992), or N stage (*p* = 0.235) (Table 1).

**Table 1:** Clinicopathological findings of two groups stratified by CA19-9 change rate (α).

**Variables**	**0.2 ≤ α ≤ 0.2 (*N* = 312)**	**α < −0.2 or α>0.2 (*N* = 341)**	***p***
Gender			0.353
Male	210 (67.3)	241 (70.7)	
Female	102 (32.7)	100 (29.3)	
Age			0.004
<60	174 (55.8)	152 (44.4)	
≥60	138 (44.2)	189 (55.4)	
Extent of resection			0.785
Distal gastrectomy	193 (61.9)	207 (60.9)	
Total gastrectomy	86 (27.6)	92 (27.0)	
Proximal gastrectomy	33 (10.6)	42 (12.3)	
Tumor location			0.992
Upper	71 (22.8)	81 (23.8)	
Middle	31 (9.9)	33 (9.7)	
Lower	178 (57.1)	193 (56.6)	
Whole	32 (10.3)	34 (10.0)	
Tumor size			0.004
<2cm	65 (20.8)	41 (12.0)	
2-5cm	162 (51.9)	173 (50.7)	
5-8cm	69 (22.1)	99 (29.0)	
>8cm	16 (5.1)	28 (8.2)	
Macroscopic type			0.012
0-II	211 (67.6)	198 (58.1)	
III-IV	101 (32.4)	143 (41.9)	
Histological grade			0.038
G1/G2	101 (32.4)	137 (40.2)	
G3/G4	211 (67.6)	204 (59.8)	
T stage			<0.001
T1	93 (29.8)	53 (15.5)	
T2	66 (21.2)	48 (14.1)	
T3	57 (18.3)	71 (20.8)	
T4a	84 (26.9)	146 (42.8)	
T4b	12 (3.8)	23 (6.7)	
N stage			0.235
N0	121 (38.8)	121 (35.5)	
N1	58 (18.6)	56 (16.4)	
N2	64 (20.5)	61 (17.9)	
N3a	46 (14.7)	69 (20.2)	
N3b	23 (7.4)	34 (10.0)	
TNM stage			<0.001
I	110 (35.3)	71 (20.8)	
II	79 (25.3)	92 (27.0)	
III	123 (39.4)	178 (52.2)	
Adjuvant chemotherapy			0.305
No	158 (50.6)	159 (46.6)	
Yes	154 (49.4)	182 (53.4)	

*G1, well differentiated; G2, moderately differentiated; G3, poorly differentiated; G4, undifferentiated; α, CA199 change rate*.

As demonstrated in Table 2, logistic regression analyses were performed to determine the risk factors for CA19-9 change. In the univariate analyses, the involved variables significantly consisted of clinicopathologic factors: macroscopic type (*p* = 0.012), age (*p* = 0.004), histological grade (*p* = 0.039), and T stage (*p* < 0.001). Multivariate analyses found that age (*p* = 0.004), histological grade (*p* = 0.039), and T stage (*p* < 0.001) were independent risk factors for CA19-9 change.

**Table 2:** Logistic regression analysis of the risk factors for CA19-9 change.

	**Univariate analysis**		**Multivariate analysis**	
**Factors**	**OR (95% CI)**	***P***	**OR (95% CI)**	***P***
Macroscopic type		0.012		
0-II	1.000			
III-IV	1.509 (1.095–2.078)			
Age		0.004		0.039
<60	1.000		1.000	
≥60	1.568 (1.151–2.136)		1.407 (1.018–1.945)	
Histological grade		0.039		0.007
G1/G2	1.000		1.000	
G3/G4	0.713 (0.517–0.983)		0.621 (0.439–0.877)	
T stage		<0.001		<0.001
T1	1.000		1.000	
T2	1.276 (0.772–2.108)	0.341	1.346 (0.809–2.239)	0.252
T3	2.186 (1.346–3.550)	0.002	2.229 (1.363–3.647)	0.001
T4a	3.050 (1.982–4.693)	<0.001	3.408 (2.182–5.332)	<0.001
T4b	3.363 (1.549–7.302)	0.002	3.645 (1.659–8.009)	0.001

*CI, confidence interval; OR, odds ratio*.

### Prognostic Significance of CA19-9 Change Before PSM

**Figure 2:**
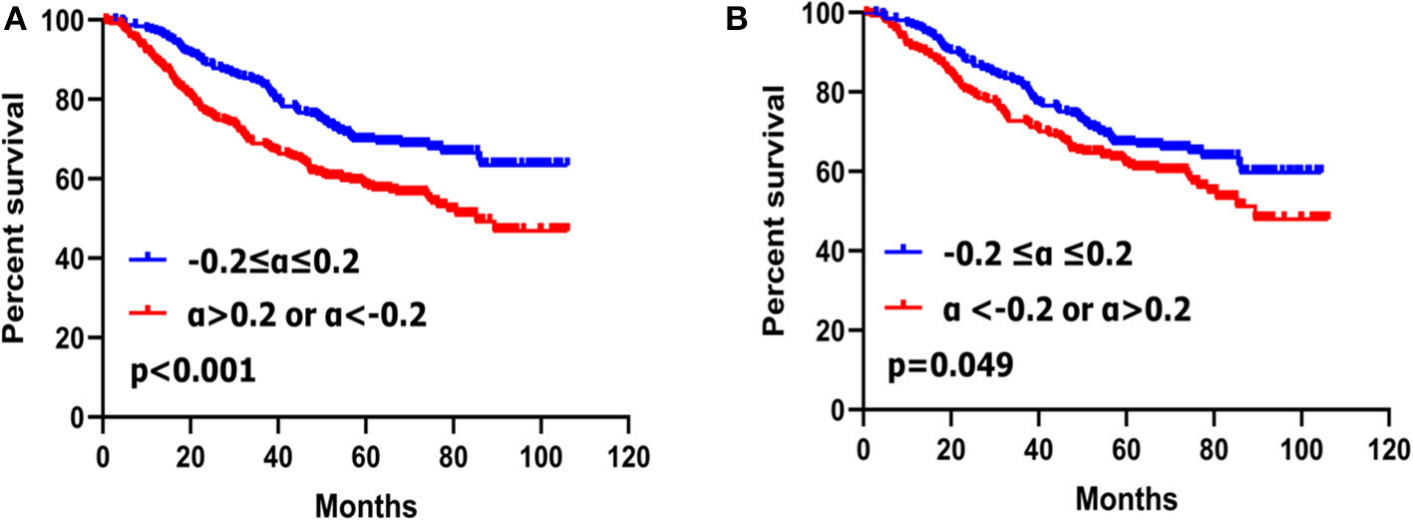
Kaplan–Meier survival analyses of two subgroups of gastric cancer patients with CA19-9 change before PSM **(A)** and after PSM **(B)**.

Patients with the CA19-9 change no more than 20% had better survival than those with CA19-9 change more than 20% (*p* < 0.001) (Figure 2A).

Univariate analysis showed that CA19-9 change (*p* < 0.001), tumor location (*p* = 0.002), histological grade (*p* = 0.002), macroscopic type (*p* < 0.001), tumor size (*p* < 0.001), extent of resection (*p* = 0.021), T stage (*p* < 0.001), N stage (*p* < 0.001), and TNM stage (*p* < 0.001) were closely associated with overall survival. Multivariate analysis with Cox regression indicated that CA19-9 change (*p* = 0.001) and other clinicopathological factors such as histological grade (*p* = 0.048) and TNM stage (*p* < 0.001) were independent prognostic factors for gastric cancer (Supplementary Table 3).

### Prognostic Significance of CA19-9 Change After PSM

To balance the baseline and reduce the impact of potential confounding factors and selection bias, we used 1:1 PSM to adjust for age, tumor size, macroscopic type, histological grade, and T stage and TNM stage between patients with the CA19-9 change no more than 20% and more than 20%. After matching, all clinicopathologic features were found to be balanced (all *p* > 0.05) between the two groups (Table 3).

**Table 3:** Clinicopathological findings of two groups stratified by CA19-9 change rate (α) after PSM.

**Variables**	**−0.2 ≤ α ≤ 0.2 (*N* = 260)**	**α < −0.2 or α > 0.2 (*N* = 260)**	***P***
Gender			0.926
Male	175 (67.3)	174 (66.9)	
Female	85 (32.7)	86 (33.1)	
Age			0.861
<60	134 (51.5)	132 (50.8)	
≥60	126 (48.5)	128 (49.2)	
Extent of resection			0.497
Distal gastrectomy	154 (59.2)	163 (62.7)	
Total gastrectomy	79 (30.4)	67 (25.8)	
Proximal gastrectomy	27 (10.4)	30 (11.5)	
Tumor location			0.678
Upper	65 (25.0)	57 (21.9)	
Middle	24 (9.2)	26 (10.0)	
Lower	144 (55.4)	155 (59.6)	
Whole	27 (10.4)	22 (8.5)	
Tumor size			0.688
<2 cm	49 (18.8)	40 (15.4)	
2–5 cm	129 (49.6)	139 (53.5)	
5–8 cm	67 (25.8)	64 (24.6)	
>8 cm	15 (5.8)	17 (6.5)	
Macroscopic type			0.854
0-II	167 (64.2)	169 (65.0)	
III-IV	93 (35.8)	91 (35.0)	
Histological grade			0.111
G1/G2	76 (29.2)	93 (35.8)	
G3/G4	184 (70.8)	167 (64.2)	
T stage			0.332
T1	49 (18.8)	53 (20.4)	
T2	63 (24.2)	45 (17.3)	
T3	52 (20.0)	59 (22.7)	
T4a	84 (32.3)	94 (36.2)	
T4b	12 (4.6)	9 (3.5)	
N stage			0.125
N0	87 (33.5)	102 (39.2)	
N1	41 (15.8)	45 (17.3)	
N2	63 (24.2)	39 (15.0)	
N3a	46 (17.7)	50 (19.2)	
N3b	23 (8.8)	24 (9.2)	
TNM stage			0.912
I	66 (25.4)	70 (26.9)	
II	72 (27.7)	72 (27.7)	
III	122 (46.9)	118 (45.4)	
Adjuvant chemotherapy			0.726
No	127 (48.8)	123 (47.3)	
Yes	133 (51.2)	137 (52.7)	

*CI, Confidence Interval; G1, well differentiated; G2, moderately differentiated; G3, poorly differentiated; G4, undifferentiated; α, CA199 change rate*.

Patients with CA19-9 changes no more than 20% had better survival rate than those with CA19-9 change more than 20% (*p* = 0.049) (Figure 2B) after matching.

Univariate analysis showed that CA19-9 change (*p* = 0.049), gender (*p* = 0.021), tumor location (*p* = 0.010), histological grade (*p* = 0.001), macroscopic type (*p* = 0.002), tumor size (*p* < 0.001), extent of resection (*p* = 0.042), T stage (*p* < 0.001), N stage (*p* < 0.001), TNM stage (*p* < 0.001) were closely associated with overall survival of gastric cancer patients. Multivariate analysis with Cox regression indicated that CA19-9 change (*p* = 0.010), gender (*p* = 0.031), histological grade (*p* = 0.036) and TNM stage (*p* < 0.001) were independent prognostic factors for gastric cancer (Table 4).

**Table 4:** Univariate and multivariate analysis of prognostic factors in gastric cancer patients using the cox proportional hazards model after PSM.

**Variables**	**Univariate analysis**			**Multivariate analysis**		
	**HR**	**95% CI**	***p***	**HR**	**95% CI**	***p***
Gender						
Male	1.000			1.000		
Female	1.400	1.053–1.862	0.021	1.385	1.030–1.863	0.031
Age						
<60	1.000					
≥60	0.604	0.703–1.228	0.929			
CA19-9 change						
−0.2 ≤ α ≤ 0.2	1.000			1.000		
α < −0.2 or α > 0.2	1.323	1.000–1.751	0.049	1.451	1.093–1.928	0.010
Tumor size			<0.001			0.744
<2 cm	1.000			1.000		
2–5 cm	1.612	1.013–2.565	0.044	1.089	0.660–1.796	0.738
5–8 cm	2.482	1.527–4.034	<0.001	1.257	0.718–2.201	0.424
>8 cm	3.697	2.000–6.835	<0.001	1.384	0.677–2.828	0.373
Extent of resection			0.042			0.555
Distal gastrectomy	1.000			1.000		
Total gastrectomy	1.432	1.055–1.943	0.021	1.288	0.799–2.076	
Proximal gastrectomy	0.899	0.558–1.446	0.660	1.116	0.583–2.134	
Tumor location			0.010			0.098
Upper	1.000			1.000		
Middle	0.702	0.394–1.253	0.231	0.720	0.387–1.340	0.300
Lower	0.876	0.625–1.229	0.443	1.074	0.630–1.833	0.792
Whole	1.707	1.067–2.733	0.026	1.601	0.930–2.754	0.089
Macroscopic type						
0-II	1.000			1.000		
III-IV	1.558	1.176–2.065	0.002	0.989	0.716–1.366	0.947
Histological grade						
G1/G2	1.000			1.000		
G3/G4	1.711	1.236–2.369	0.001	1.440	1.024–2.024	0.036
T stage			<0.001			
T1	1.000					
T2	1.514	0.883–2.597	0.132			
T3	1.805	1.070–3.044	0.027			
T4a	3.394	2.131–5.405	<0.001			
T4b	2.907	1.376–6.142	<0.001			
N stage			<0.001			
N0	1.000					
N1	1.561	0.976–2.495	0.063			
N2	1.891	1.232–2.902	0.004			
N3a	3.334	2.230–4.984	<0.001			
N3b	5.494	3.484–8.662	<0.001			
TNM stage			<0.001			<0.001
I	1.000			1.000		
II	1.855	1.149–2.995	0.011	1.746	1.045–2.917	0.033
III	3.780	2.475–5.771	<0.001	3.029	1.844–4.976	<0.001
Adjuvant chemotherapy						
No	1.000					
Yes	0.883	0.668–1.167	0.381			

*HR, Hazard Ratio; CI, Confidence Interval; G1, well differentiated; G2, moderately differentiated; G3, poorly differentiated; G4, undifferentiated; α, CA199 change rate*.

**Figure 3:**
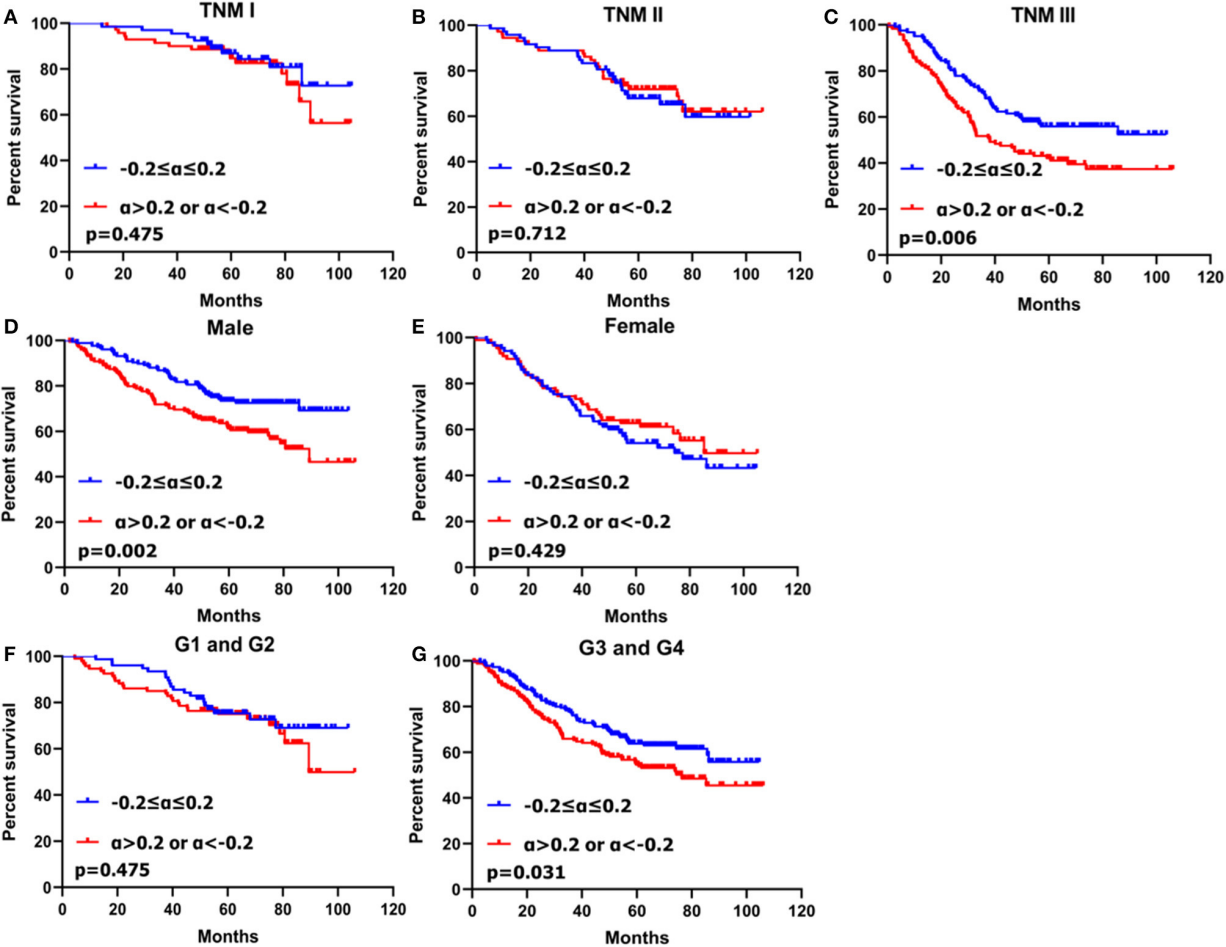
Kaplan–Meier survival analyses of two subgroups of gastric cancer patients with CA19-9 change stratified by TNM stage **(A–C)**, gender **(D,E)** and histological grade **(F,G)**.

In stratification analysis based on gender, histological grade and TNM stage, patients with CA19-9 change more than 20% had worse prognosis that those with CA19-9 change no more than 20% in male (*p* = 0.002), poorly differentiated or undifferentiated adenocarcinoma (*p* = 0.031) and TNM stage III (*p* = 0.006) (Figure 3).

## Discussion

The serum tumor marker CA19-9 have been widely applied in gastrointestinal malignancies. To date, a series of studies have explored the diagnostic and prognostic value of serum tumor markers including CA19-9 in gastric cancer (10, 13, 14). Elevated preoperative serum markers are generally associated with tumor progression, and most reports concluded that preoperative elevated serum markers such as CA19-9 and CEA were significantly associated with poor long-term patient survival (4, 9, 20). Postoperative CA19-9 levels have been utilized not only for detecting recurrence early but also for the prediction of survival of gastric cancer patients (14). Nevertheless, the prognostic significance of CA19-9 remains controversial and requires further investigation because of its low positivity rate, low sensitivity and high false-positive rates. For these reasons, analysis of changes in levels of preoperative and postoperative serum markers may provide a new way for examining the prognostic significance of serum CA19-9 levels in gastric cancer. Previous studies suggested that the half-life of CA19-9 was short and the level of serum tumor marker would change over time (21, 22). In this manner, the level of a serum tumor marker might be change from before to after surgery. Most previous studies focused only on preoperative or postoperative values of serum CA19-9. In this study, we explored the prognostic value of serum tumor marker CA19-9 change before and after gastrectomy. To the best of our knowledge, ours is the first study to report the prognostic impact of serum CA19-9 change before and after curative resection for gastric cancer patients.

We found that CA19-9 change significantly correlated with age, tumor size, macroscopic type, histological grade, T stage and TNM stage. Gastric cancer patients with CA19-9 changes <20% had significant better overall survival than those with change more than 20%; Cox regression analysis revealed the CA19-9 change was an independent risk factor for survival before and after PSM.

A strong correlation between elevated tumor marker CA19-9 and clinicopathological features has already been reported in gastric cancer. Those studies have demonstrated that preoperative serum CA19-9 was significantly associated with tumor size, differentiated tumor types, the tumor depth, nodal involvement, peritoneal metastases and TNM stage, and elevated preoperative serum CA19-9 was associated with aggressive tumor behavior (23–25). In the present study, CA19-9 change significantly correlated with tumor size, macroscopic type, histological grade, tumor depth, and TNM stage. Patients with CA19-9 change more than 20% had larger tumor size, more macroscopic type III–IV, deeper tumor invasion, and more advanced TNM stage than those with CA19-9 change <20%. It appears that CA19-9 with changes more than 20% were similar to elevated preoperative serum CA19-9 that corelated with aggressive tumor behavior. However, gastric cancer patients with CA19-9 change more than 20% were older and had less poorly differentiated tumor types and no association was found between CA19-9 change and nodal involvement in this study, which differed from results of previous reports (23–25). These results indicated that preoperative serum CA19-9 level and CA19-9 change might have different clinical significance. Another reason might be postoperative serum CA19-9 level had some effects on the relationship between preoperative serum CA19-9 and nodal involvement. Logistic regression analyses in our study suggested that age, histological grade and T stage were independent risk factors for CA19-9 change, suggesting that these factors were closely associated with CA19-9 change.

Whether serum CA19-9 levels should be used as prognostic indicators in patients with gastric cancer has long been discussed; however, the results remain controversial. Some studies reported that CA19-9 level was an independent prognostic factor by multivariate analysis in gastric cancer; gastric cancer patients with elevated CA19-9 levels had poorer prognosis than those with normal CA19-9 (13, 14, 16, 26). However, other studies showed than preoperative CA19-9 levels were not an independent prognostic factor in gastric cancer (27, 28). In the present study, we found that CA19-9 change was an independent prognostic factor before PSM. We also found that there might exist multicollinearity with CA19-9 change, and this could be a potential confounding factor for CA19-9 change. Therefore, propensity score matching was applied to reduce the multicollinearity and to balance the baseline. After matching, CA19-9 change was found to be an independent predictor, and patients with CA19-9 change more than 20% had worse survival than those with CA19-9 change <20%.

Shimada et al. reported that the positive rates for preoperative CA19-9 of gastric cancer increased with TNM stage (14). Some authors reported that postoperative CA19-9 levels increased for the first time at recurrence, and that CA19-9 may be particularly useful as a marker of peritoneal recurrence (29, 30). Tumor markers were expressed in the process of tumorigenesis and progression and elevated serum tumor marker levels may indicate the presence of a neoplasm as well as the relative high malignancy burden and aggressive nature of the biological response (31). For patients with CA19-9 decreasing more than 20%, they might have higher tumor burdens before surgery; therefore, the substantial decrease might reflect the efficiency of radical operation and an elimination of the invasive potential of the cancer. However, those patients with higher tumor burdens may be more prone to relapse, resulting in a poor prognosis. For patients with CA19-9 increasing more than 20%, the large increase might indicate the potential for the increased prevalence micro-metastases. After PSM, further stratification analysis indicated that patients with CA19-9 change more than 20% had worse prognosis that those with CA19-9 change no more than 20% in male, poorly differentiated or undifferentiated adenocarcinoma and TNM stage III. Those patients might more likely to have recurrence after surgery. Our result suggested that clinicians ought to focus attention on gastric cancer patients with CA19-9 changes more than 20%, especially those in male, poorly differentiated or undifferentiated adenocarcinoma and TNM stage III, as those patients might require closer postoperative follow-up.

This study had some limitations: First, there was possible selection bias and performance of analysis bias because it was a retrospective study. Second, we only studied the prognostic value of serum CA19-9 change; other serum tumor markers such as CEA, CA72-4 and CA125 need to be further investigated. Finally, we reported the prognostic value of serum CA19-9 changes in our institution and a large-scale, well-designed prospective study is needed in the future.

In conclusion, changes in preoperative and postoperative serum CA19-9 levels were closely associated with clinicopathological traits and was an independent prognostic factor in gastric cancer patients, which may be regarded as reliable markers for survival prediction.

## Data Availability Statement

The datasets generated for this study are available on request to the corresponding author.

## Ethics Statement

This study was approved by the Research Ethics Committee of West China Hospital.

## Author Contributions

J-KH contributed to the conception and quality control of the study. X-HS, KL, S-JY, and W-HZ contributed significantly to analysis and manuscript preparation. X-HS, X-LC, and L-YZ performed the data analyses and wrote the manuscript. Z-GZ, X-ZC, and KY helped to perform the analysis with constructive discussions. All authors contributed to the article and approved the submitted version.

## Conflict of Interest

The authors declare that the research was conducted in the absence of any commercial or financial relationships that could be construed as a potential conflict of interest.

## Abbreviations

G1: well-differentiated
G2: moderately differentiated
G3: poorly differentiated
G4: undifferentiated
HR: hazard ratio
CI: confidence interval.
