# Supplementary material for: Prognostic Value of Changes in Preoperative and Postoperative Serum CA19-9 Levels in Gastric Cancer
Source: Front Oncol. 2020 Aug 18;10:1432. doi: 10.3389/fonc.2020.01432 (PMC7461783; doi:10.3389/fonc.2020.01432)
Supplement: Supplementary file 1 [file Table_1.docx]

| Supplementary Table 1: Clinicopathological findings of gastric cancer patients with CA199 decreasing and increasing less than 20% | | | |
| --- | --- | --- | --- |
| Variables | -0.2≤α≤0  (N=125) | 0<α≤0.2  (N=187) | p |
| Gender |  |  | 0.831 |
| Male | 85(68.0) | 125(66.8) |  |
| Female | 40(32.0) | 62(33.2) |  |
| Age |  |  | 0.947 |
| <60 | 70(56.0) | 104(55.6) |  |
| ≥60 | 55(44.0) | 83(44.2) |  |
| Extent of resection |  |  | 0.857 |
| Distal gastrectomy | 75(60.0) | 118(63.1) |  |
| Total gastrectomy | 36(28.8) | 50(26.7) |  |
| Proximal gastrectomy | 14(11.2) | 19(10.2) |  |
| Tumor location |  |  | 0.564 |
| Upper | 26(20.8) | 45(24.1) |  |
| Middle | 14(11.2) | 17(9.1) |  |
| Lower | 75(60.0) | 103(55.1) |  |
| Whole | 10(8.0) | 22(11.8) |  |
| Tumor size |  |  | 0.607 |
| <2cm | 28(22.4) | 37(19.8) |  |
| 2-5cm | 68(54.4) | 94(50.3) |  |
| 5-8cm | 23(18.4) | 46(24.6) |  |
| >8cm | 6(4.8) | 10(5.3) |  |
| Macroscopic type |  |  | 0.177 |
| 0-II | 90 (72.0) | 121(64.7) |  |
| III-IV | 35(28.0) | 66(35.3) |  |
| Histological grade |  |  | 0.531 |
| G1/G2 | 43(34.4) | 58(31.0) |  |
| G3/G4 | 82(65.6) | 129(69.0) |  |
| T stage |  |  | 0.638 |
| T1 | 32(25.6) | 61(32.6) |  |
| T2 | 30(24.0) | 36(19.3) |  |
| T3 | 23(18.4) | 34(18.2) |  |
| T4a | 36(28.8) | 48(25.7) |  |
| T4b | 4(3.2) | 8(4.3) |  |
| N stage |  |  | 0.756 |
| N0 | 49(39.2) | 72(38.5) |  |
| N1 | 19(15.2) | 39(20.9) |  |
| N2 | 28(22.4) | 36(19.3) |  |
| N3a | 20(16.0) | 26(13.9) |  |
| N3b | 9(7.2) | 14(7.5) |  |
| TNM stage |  |  | 0.654 |
| I | 41(32.8) | 69(36.9) |  |
| II | 31(24.8) | 48(25.7) |  |
| III | 53(42.4) | 70(37.4) |  |
| Adjuvant chemotherapy |  |  | 0.695 |
| No | 64(52.0) | 93(49.7) |  |
| Yes | 60(48.0) | 94(50.3) |  |
| G1= well differentiated; G2 = moderately differentiated; G3 = poorly differentiated; G4 = undifferentiated; α=CA199 change rate | | | |
