# Supplementary material for: Prognostic Value of Changes in Preoperative and Postoperative Serum CA19-9 Levels in Gastric Cancer
Source: Front Oncol. 2020 Aug 18;10:1432. doi: 10.3389/fonc.2020.01432 (PMC7461783; doi:10.3389/fonc.2020.01432)
Supplement: Supplementary file 2 [file Table_2.docx]

| Supplementary Table 2: Clinicopathological findings of gastric cancer patients with CA199 decreasing and increasing more than 20% | | | |
| --- | --- | --- | --- |
| Variables | α<-0.2  (N=165) | α>0.2  (N=176) | p |
| Gender |  |  | 0.570 |
| Male | 119(72.1) | 122(69.3) |  |
| Female | 46(27.9) | 54(30.7) |  |
| Age |  |  | 0.439 |
| <60 | 70(42.4) | 82(46.6) |  |
| ≥60 | 95(57.6) | 94(53.4) |  |
| Extent of resection |  |  | 0.825 |
| Distal gastrectomy | 102(61.8) | 105(59.7) |  |
| Total gastrectomy | 42(25.5) | 50(28.4) |  |
| Proximal gastrectomy | 21(12.7) | 21(11.9) |  |
| Tumor location |  |  | 0.938 |
| Upper | 40(24.2) | 41(23.3) |  |
| Middle | 17(10.3) | 16(9.1) |  |
| Lower | 93(56.4) | 100(56.8) |  |
| Whole | 15(9.1) | 19(10.8) |  |
| Tumor size |  |  | 0.919 |
| <2cm | 19(11.5) | 22(12.5) |  |
| 2-5cm | 85(51.5) | 88(50.0) |  |
| 5-8cm | 49(29.7) | 50(28.4) |  |
| >8cm | 12(7.3) | 16(9.1) |  |
| Macroscopic type |  |  | 0.793 |
| 0-II | 97(58.8) | 101(57.4) |  |
| III-IV | 68(41.2) | 75(42.6) |  |
| Histological grade |  |  | 0.412 |
| G1/G2 | 70(42.4) | 67(38.1) |  |
| G3/G4 | 95(57.6) | 109(61.9) |  |
| T stage |  |  | 0.055 |
| T1 | 27(16.4) | 26(14.8) |  |
| T2 | 32(19.4) | 16(9.1) |  |
| T3 | 28(17.0) | 43(24.4) |  |
| T4a | 68(41.2) | 78(44.3) |  |
| T4b | 10(6.1) | 13(7.4) |  |
| N stage |  |  | 0.147 |
| N0 | 69(41.8) | 52(29.5) |  |
| N1 | 25(15.2) | 31(17.6) |  |
| N2 | 27(16.4) | 34(19.3) |  |
| N3a | 32(19.4) | 37(21.0) |  |
| N3b | 12(7.3) | 22(12.5) |  |
| TNM stage |  |  | 0.051 |
| I | 38(23.0) | 33(18.8) |  |
| II | 52(31.5) | 40(22.7) |  |
| III | 75(45.5) | 103(58.5) |  |
| Adjuvant chemotherapy |  |  | 0.654 |
| No | 79(47.9) | 80(45.5) |  |
| Yes | 86(52.1) | 96(54.5) |  |
| G1= well differentiated; G2 = moderately differentiated; G3 = poorly differentiated; G4 = undifferentiated; α=CA199 change rate | | | |
