# Supplementary material for: Prognostic Value of Changes in Preoperative and Postoperative Serum CA19-9 Levels in Gastric Cancer
Source: Front Oncol. 2020 Aug 18;10:1432. doi: 10.3389/fonc.2020.01432 (PMC7461783; doi:10.3389/fonc.2020.01432)
Supplement: Supplementary file 3 [file Table_3.docx]

| Supplementary Table 3: Univariate and multivariate analysis of prognostic factors in gastric cancer patients before PMS using the cox proportional hazards model | | | | | | |
| --- | --- | --- | --- | --- | --- | --- |
|  | Univariate analysis | | | Multivariate analysis | | |
|  | HR | 95% CI | p | HR | 95% CI | p |
| Gender |  |  |  |  |  |  |
| Male | 1.000 |  |  | 1.000 |  |  |
| Female | 1.220 | 0.941-1.582 | 0.134 |  |  |  |
| Age |  |  |  |  |  |  |
| <60 | 1.000 |  |  | 1.000 |  |  |
| ≥60 | 0.982 | 0.767-1.258 | 0.885 |  |  |  |
| CA19-9 change |  |  |  |  |  |  |
| -0.2≤α≤0.2 | 1.000 |  |  | 1.000 |  |  |
| α<-0.2 or α>0.2 | 1.639 | 1.272-2.113 | <0.001 | 1.543 | 1.190-2.000 | 0.001 |
| Tumor size |  |  | <0.001 |  |  | 0.906 |
| <2cm | 1.000 |  |  | 1.000 |  |  |
| 2-5cm | 1.802 | 1.162-2.794 | 0.009 | 1.087 | 0.676-1.746 | 0.731 |
| 5-8cm | 2.894 | 1.837-4.560 | <0.001 | 1.194 | 0.704-2.025 | 0.510 |
| >8cm | 3.714 | 2.121-6.506 | <0.001 | 1.135 | 0.591-2.177 | 0.704 |
| Extent of resection |  |  | 0.021 |  |  | 0.989 |
| Distal gastrectomy | 1.000 |  |  | 1.000 |  |  |
| Total gastrectomy | 1.465 | 1.114-1.927 | 0.006 | 1.023 | 0.693-1.512 | 0.907 |
| Proximal gastrectomy | 1.029 | 0.690-1.535 | 0.888 | 0.995 | 0.587-1.688 | 0.986 |
| Tumor location |  |  | 0.002 |  |  | 0.081 |
| Upper | 1.000 |  |  | 1.000 |  |  |
| Middle | 0.787 | 0.484-1.278 | 0.333 | 0.889 | 0.527-1.500 | 0.659 |
| Lower | 0.803 | 0.594-1.085 | 0.153 | 0.896 | 0.573-1.401 | 0.630 |
| Whole | 1.601 | 1.067-2.404 | 0.023 | 1.490 | 0.927-2.396 | 0.099 |
| Macroscopic type |  |  |  |  |  |  |
| 0-II | 1.000 |  |  | 1.000 |  |  |
| III/IV | 1.705 | 1.330-2.185 | <0.001 | 1.048 | 0.790-1.389 | 0.747 |
| Histological grade |  |  |  |  |  |  |
| G1/G2 | 1.000 |  |  | 1.000 |  |  |
| G3/G4 | 1.523 | 1.161-1.999 | 0.002 | 1.334 | 1.003-1.773 | 0.048 |
| T stage |  |  | <0.001 |  |  |  |
| T1 | 1.000 |  |  | 1.000 |  |  |
| T2 | 1.612 | 0.982-2.646 | 0.059 |  |  |  |
| T3 | 1.912 | 1.197-3.056 | 0.007 |  |  |  |
| T4a | 3.817 | 2.545-5.724 | <0.001 |  |  |  |
| T4b | 4.383 | 2.478-7.752 | <0.001 |  |  |  |
| N stage |  |  | <0.001 |  |  |  |
| N0 | 1.000 |  |  | 1.000 |  |  |
| N1 | 1.601 | 1.057-2.426 | 0.026 |  |  |  |
| N2 | 2.387 | 1.642-3.470 | <0.001 |  |  |  |
| N3a | 3.423 | 2.375-4.932 | <0.001 |  |  |  |
| N3b | 6.005 | 3.987-9.046 | <0.001 |  |  |  |
| TNM stage |  |  | <0.001 |  |  | <0.001 |
| I | 1.000 | 1 |  | 1.000 |  |  |
| II | 1.921 | 1.248-2.958 | 0.003 | 1.681 | 1.056-2.676 | 0.028 |
| III | 4.228 | 2.906-6.152 | <0.001 | 3.280 | 2.098-5.129 | <0.001 |
| Adjuvant chemotherapy |  |  |  |  |  |  |
| No | 1.000 |  |  |  |  |  |
| Yes | 0.954 | 0.744-1.222 | 0.707 |  |  |  |
| HR: Hazard Ratio; CI: Confidence Interval; G1= well differentiated; G2 = moderately differentiated; G3 = poorly differentiated; G4 = undifferentiated; α=CA199 change rate | | | | | | |
